# Impact of Dental Midline Shift on the Perception of Facial Attractiveness in Young Adults

**DOI:** 10.3390/jcm13133944

**Published:** 2024-07-05

**Authors:** Babak Sayahpour, Sara Eslami, Ralf Usherenko, Abdolreza Jamilian, Mauricio Gonzalez Balut, Nicolas Plein, Vincenzo Grassia, Ludovica Nucci

**Affiliations:** 1 Department of Orthodontics, Johann-Wolfgang Goethe University, 60596 Frankfurt am Main, Germany; sayahpour@med.uni-frankfurt.de (B.S.); plein@med.uni-frankfurt.de (N.P.); 2 Private Dental Practice, 63071 Offenbach, Germany; ralf_u@outlook.com; 3 Department of Orthodontics, Dental School, Cranio-Maxillofacial Research Center, Tehran Islamic Azad University of Medical Sciences, Tehran 19395-1495, Iran; info@jamilian.net; 4 City of London Dental School, University of Bolton, Bolton BL3 5AB, UK; 5 Department of Orthodontics and Dentofacial Orthopedics, Loma Linda University, San Bernadino, CA 92408, USA; mgbalut@hotmail.com; 6 Multidisciplinary Department of Medical-Surgical and Dental Specialties, University of Campania Luigi Vanvitelli, 80131 Naples, Italy; grassiavincenzo@libero.it (V.G.); ludortho@gmail.com (L.N.)

**Keywords:** dental midline shift, smile attractiveness, dental aesthetics, dentofacial symmetry, smile symmetry

## Abstract

**Background/Objectives:** The aim of this study was to quantify the threshold of a dental midline shift that would compromise facial attractiveness and indicate a need for treatment from the points of view of laypeople and dental professionals. **Methods**: Whole-face natural photographs of a male and a female model were digitally manipulated to create various degrees of upper and lower dental midline shifts through bodily movement of the upper or lower midlines as well as alteration of the axial inclination of the upper teeth. The samples were then assessed by two groups of observers (laypeople (LP) and dental professionals (DP)). **Results**: The lower midline shift did not negatively affect the DP and LP’s perceptions of smile attractiveness. The first significant loss of attractiveness was registered by the DP with an upper midline shift of 1 mm in the female model. However, the LP registered this at 2 mm. The DP registered the necessity of treatment at a threshold of 2 mm in the female model and 3 mm in the male model. LP identified the need for treatment at 3 mm for both males and females. The female model was judged more critically than her male counterpart by both female and male observers. **Conclusions**: DP assess the midline deviation more critically than LP. Both DP and LP were more sensitive to midline deviations in the female model regardless of their own gender. Both groups registered the need for treatment at a higher threshold than the reduction in smile attractiveness.

## 1. Introduction

In recent years, aesthetics have become increasingly more important in people’s social perception. Consequently, this development is also reflected and represented in dentistry and orthodontics. An aesthetic and harmonious smile is an important factor in the success of dental therapy [1]. This is often of utmost importance to young adult patients, who may base the success of their treatment largely on the result of the soft tissue profile and the attractiveness of their smile [2]. One of the most important aspects of facial aesthetics and the perceived attractiveness of a smile is the correspondence of the midlines between the face and teeth. This aspect has been acknowledged and analyzed in numerous studies [3,4,5,6,7,8,9,10,11]. Ideally, the centers of the face and the dental arch should be congruent to each other [12]; when this is not the case, it can be defined as a midline shift (MLS). While MLS is commonly discussed in the context of smile aesthetics, its potential functional implications as a causal factor in temporomandibular disorders (TMDs) warrant further attention. The association between MLS and craniomandibular disorders has been a topic of debate, with current evidence being insufficient and inconclusive [13]. Some studies have identified significant associations [14,15], while others have not [16], suggesting that the causal role of MLS in TMDs cannot be definitively excluded. Therefore, exploring MLS from a functional perspective is essential.

Nevertheless, the presence of asymmetries such as a midline shift is more the rule than the exception and the prevalence of such dentofacial asymmetries is about 80% among patients [17,18,19]. A lack of perfect symmetry in a patient’s face could mask their dental midline shift or aggravate its apparent severity [20]. In most of these studies, a perfectly symmetric face is constructed digitally, and the midline shifts are generated by altering this ideal symmetric base image. Other studies use cropped images, eliminating the patient’s hair, forehead, eyes, and ears. However, the digital construction of a perfectly symmetric face not only leads to the loss of preservation of their natural smile, but it also makes the midline deviation in absence of any other asymmetries more noticeable. Using cropped images of the lower facial third or just the mouth area also leads to under- or overestimation of the effect of midline deviation on the perception of smile and facial attractiveness [21]. Since the therapy of midline discrepancies usually adds to the complexity, costs, and length of the treatment, a patient-oriented estimation of the negative impact of a midline deviation on their facial attractiveness is of utmost importance [22]. Therefore, the present study aimed to quantify the threshold of the dental midline shift in young adults that would negatively affect their facial attractiveness and necessitate a treatment.

## 2. Materials and Methods

### 2.1. Ethics Committee Approval

This study was approved by the ethics committee of J. W. Goethe University under Nr. E 172/18 (intern number 401/18).

### 2.2. Preparation of Sample Photographs

**Figure 1:**
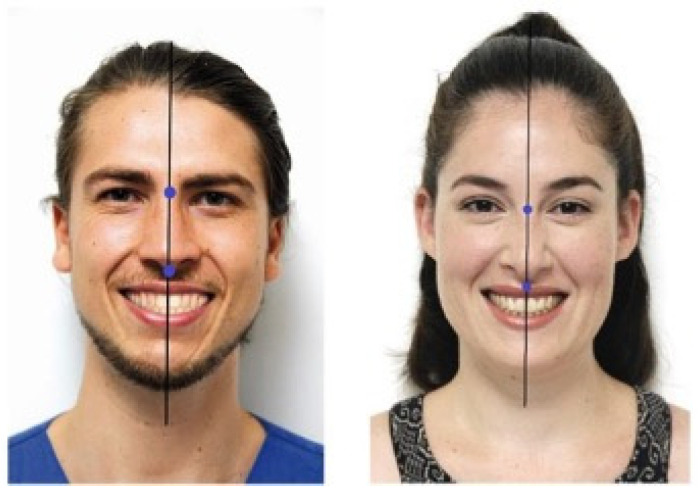
Sample photographs of a male model and a female model.

Sample photographs (Figure 1) were taken from a male and a female model who met the following criteria:

- Caucasian ethnicity;
- Young adult (between ages 25 to 35);
- Mesofacial face type;
- Skeletal and dental class I relationships with normal overjet and overbite;
- Lack of crowding, spacing, or other malalignment;
- No history of previous orthodontic or aesthetic restorative treatments;
- Lack of noticeable facial or dental asymmetries;
- Lack of noticeable deviation of the nasion, nose tip, and philtrum tip from the facial midline;
- No apparent abnormalities or defects in the teeth or dentition;
- As many visible teeth as possible when smiling;
- Naturally white teeth and lack of discolorations in the visible teeth.

### 2.3. Digital Manipulation of Sample Photographs

By using the image processing program Adobe Photoshop (CS5; Adobe Systems, San Jose, CA, USA), an optical central axis was defined for both initial images. This was based on the nasion, the nose tip, and the philtrum tip.

**Figure 2:**
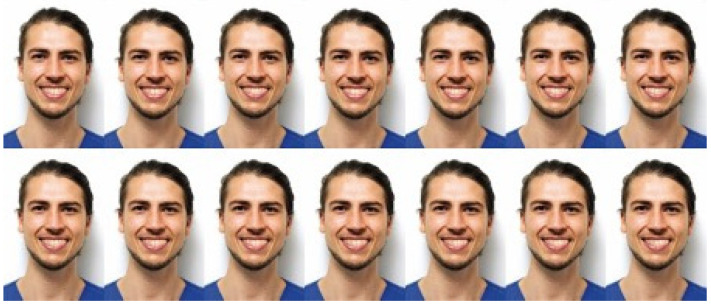
Increases of 1 mm in the shift of the midline to the right and to the left on a male model.

**Figure 3:**
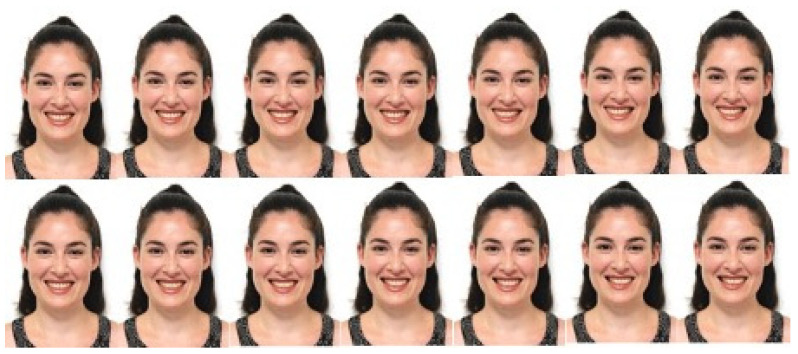
Increases of 1 mm in the shift of the midline to the right and to the left on a female model.

**Figure 4:**
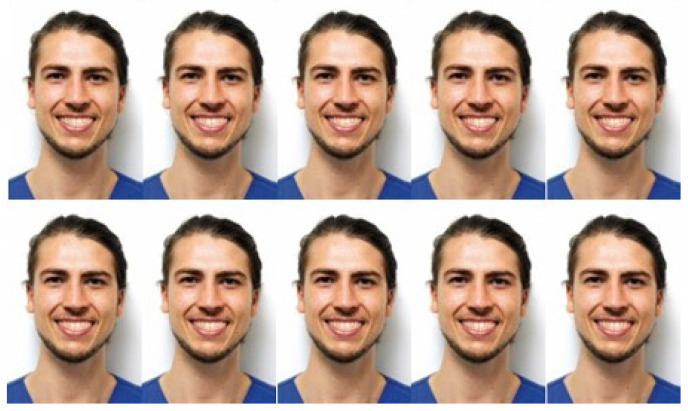
Displacement of the midline in relation to the gradual both mesial and distal modification of the axes of the upper teeth on a male model.

**Figure 5:**
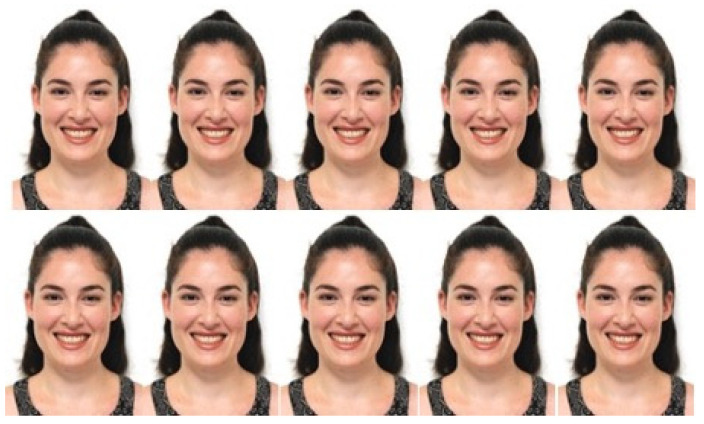
Displacement of the midline in relation to the gradual both mesial and distal modification of the axes of the upper teeth on a female model.

Three types of midline shifts were then created as follows:

- In the first group (UBS), the upper dental midline was shifted bodily. This shift was then increased in 1 mm increments and in both the left and right directions up to a maximal 6 mm, resulting in 13 photographs per gender (Figure 2 and Figure 3).

- In the second group (LBS), a lower bodily midline shift was created in 1 mm increments up to a total shift of 4 mm in both directions; nine photograph samples were created for each gender.
- The midline shift in the third group (UTS) was created by gradually altering the angulation of the upper-teeth axes in 1-degree increments in the mesial and distal directions up to a maximal 4-degree shift, resulting in 9 modified photographs per gender (Figure 4 and Figure 5).

A total of 68 images were combined into a PowerPoint presentation.

### 2.4. Participants

Forty academic dental professionals (DP group) and forty volunteers with no dental training (LP group) took part in this study. All of the participants were adults of Caucasian ethnicity between the ages of 25 to 55. The DP group were dentists employed at the J. W. Goethe University and consisted of 3 periodontists, 7 orthodontists, 8 specialists in restorative dentistry, 11 prosthodontists, and 11 oral surgeons. To analyze the possible influence of professional experience, the participants in the DP group were further divided into three categories based on their years of experience: 1—up to five years, 2—more than five but less than ten years, and 3—more than ten years of professional experience. The LP participants were staff from other faculties of the university. Any participants with a history of previous or current orthodontic or aesthetic restorative dental treatments were excluded from the study. Having a family member or a close friend currently undergoing or with a history of previous aesthetic or orthodontic treatment was also considered an exclusion criterion for the LP group.

### 2.5. Assessment of the Digitally Manipulated Photos

The participants were verbally informed in advance that they would be shown a PowerPoint presentation of a series of images of a male and a female model. They were asked to rate the attractiveness of the facial expressions of the models in the images based on their personal preference. They were not informed on what sort of modification was made to the images. To ensure that the changes were not obvious, the female and male models were shown in an alternating order throughout the presentation. Furthermore, blue intermediate slides were inserted between the slides containing the modified images to ensure optical neutralization in the sequence of the images, as well as to reduce the risk of direct comparisons between the images. To check the reliability of the participants’ evaluations, the initial images of both models were faded in at regular intervals. Each photo was presented to the viewers for ten seconds. The decision to provide 10 s per slide was made based on the study by Johnston, in order to provide enough time for the evaluation without inducing fatigue or reducing the participants’ concentration [5]. It was not possible to return to the previous image. The participants were asked to rate each photo by filling out a questionnaire containing two questions:

1. How do you rate the overall attractiveness of the facial expression of this model, ranging from 1 (extremely unattractive) to 7 (extremely attractive)?
2. Do you recognize a need for a treatment in this particular image to improve their facial attractiveness? Yes/No.

### 2.6. Statistical Analysis of the Results

Sample size calculation was performed based on the study by Beyer and Lindauer [3]. They reported a standard deviation of 1 mm for LP and 0.8 mm for DP. The difference was set as midline deviation of 0.6 mm, which was registered as the lowest yet still clinically relevant amount of deviation. The alpha was set at 5% and the power was set at 80%. The sample size of 40 per each group was required to detect 0.6 mm of midline deviation with the power of 80% and alpha of 5%.

Friedman tests for repeated measures were used to compare the degrees of facial attractiveness of the samples. A two-sample *T*-test was used to compare the differences between the participants (DP vs. LP) and a Chi-square test was used to determine which images had statistically significant deviations (*p* < 0.05).

The BiAS software (version 11.10) was used to perform the data analysis.

## 3. Results

**Figure 6:**
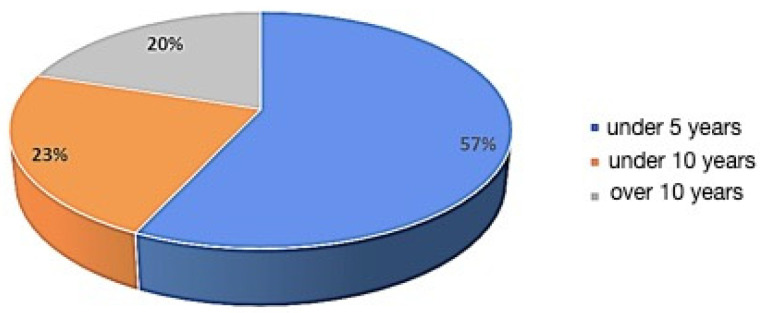
No statistically significant correlation between the gender or age of participants in both the DP and LP groups (*p* > 0.05).

Within the DP (dental professionals) and LP (laypeople) groups, no statistically significant correlations were observed between the gender or age of participants and their perceptions (*p* > 0.05). Additionally, differences in the years of experience of the DP group did not influence their perception of midline shift (Figure 6). The direction of the midline shift (MLS) did not affect the observers’ perceptions. Neither the DP nor the LP group found a lower midline shift (LBS) unattractive or in need of therapy.

The first significant decrease in attractiveness for the DP group was observed with a 1 mm upper midline shift (UBS) in the female model, while the LP group noted this decrease at 2 mm. A statistically significant difference was also found in the assessment of treatment needs for UBS in the female model. The DP group identified the need for treatment at a 2 mm threshold, whereas the LP group recognized it at 3 mm. Both the DP and LP groups recognized a need for treatment in the male model at a 3 mm threshold.

Upper midline shifts due to crown angulation alterations (UTSs) were statistically significant at 4 degrees in assessing treatment needs and were deemed unattractive by both groups. Both groups rated the attractiveness of the female face 1 mm lower than they rated the male counterpart, and midline shifts in the female model were evaluated more critically by both groups, regardless of the observer’s gender (Table 1).

The progression of the midline shift in the female model led to a steady decrease in average attractiveness scores for both groups. However, in the male model, the LP group’s attractiveness scores plateaued between 1 and 5 mm, while the DP group recorded two plateaus: one between 1 and 3 mm, and another between 4 and 5 mm. The DP group assessed overall attractiveness more critically than the LP group, with significant decreases in attractiveness occurring 1 mm earlier for both genders.

No statistical significances were noted within the DP group, suggesting a possible influence of their specialty or professional background. Notably, participants from the field of oral and maxillofacial surgery evaluated the need for treatment of midline deviations almost identically for both genders. A third of these specialists found treatment necessary for a 1 mm UBS, and 90% rated a 2 mm UBS as requiring treatment.

## 4. Discussion

The results of our study confirm that dental midline shifts significantly affect the perception of aesthetics among both dental professionals (DP) and laypeople (LP). While previous studies have primarily focused on bodily dental midline shifts, our research uniquely evaluates both bodily and angular midline shifts. This distinction is important because a midline shift can result from either the bodily shift of teeth or their mesial or distal tipping. Angular shifts are often accompanied by an occlusal cant, which can further deteriorate aesthetics. Moreover, correcting dental tippings is generally easier than correcting bodily shifts.

While some studies have reported a threshold of 4 mm in the assessment by laypeople [8,23], others have reported thresholds of 2 mm [6,9,11] or 3 mm [10]. This lack of consensus between different studies results from the different methodologies used. 

One of the major challenges lies in preparing the baseline images while preserving a natural facial expression. Some studies have opted for the digital creation of a symmetric facial model (SFM) as their baseline and the generation of asymmetric facial models (AFMs) by progressively altering the SFM [20,24]. However, the creation of an SFM leads to the loss of natural facial expression and produces bias in the mythology. Lower thresholds reported in these studies could be due to the artificial and unnatural facial expressions of the AFMs. We instead opted for the preservation of the natural appearance of the smiles and facial expressions of our models.

Many studies have used cropped images of the mouth area or the lower facial third in order to reduce the confounding factors and avoid distraction of the observers [9,25]. Removing distractors such as the nose, cheeks, and chin would increase the rating accuracy for parameters such as buccal corridors. However, the detection of midline deviation would become more complicated and lead to higher threshold values. We instead used images of the whole face of the models including their hair and neck, rather than using cropped images, in order to increase the accuracy of the ratings.

Another important strength of our study is that we included samples from both female and male models instead of using sex-neutral images [6] or images of only the female gender [20,24]. An interesting finding was the lack of any significant role of the participants’ gender or age in their perception of facial attractiveness, even though women are considered to be more critical regarding facial aesthetics [20,24]. On the contrary, both male and female observers were more critical in judging the female model’s facial attractiveness compared to her male counterpart. The exclusion of participants with a history of previous or ongoing orthodontic or aesthetic restorative treatments was another advantage of the present study, since aesthetic dental treatments could raise the observer’s consciousness in regard to smile parameters. Our evaluation was based on questionnaires filled out by the participants. 

Our results show that DP assess the midline shift more critically compared to LP. These results are in agreement with those of other studies [7]. We did not find any statistically significant differences among the dental professionals regarding their field of specialty. Nevertheless, we recorded some interesting findings on the oral and maxillofacial specialists: this group of specialists evaluated the male and female models almost identically. In contrast to our results, some studies have registered lower threshold values for orthodontists compared to specialists in other fields [7]: these thresholds have been measured at 1 mm [8], 2 mm [5], and 4 mm [23]. This could be due to the academic background of the DP group in our study, as they were selected from staff of the university. This academic background and the multi-disciplinary treatment approach in the university could have played a role in homogenizing the sensitivity of the DP group towards midline deviations. This academic background also accounts for the lack of difference regarding the years of experience in the DP group. 

We registered that an upper dental midline shift had an early effect on perceived overall attractiveness, especially in the female model. Similar findings have been reported by other studies [11]. On the contrary, shifts of the lower midline had no effects on the perceived facial attractiveness or treatment need. This could be due to the incorporation of images of young adults in this study, with mostly visible upper teeth when smiling. It would be interesting to conduct a similar study on older adults with a higher visibility of lower teeth when smiling to evaluate the effects of lower midline deviations. Another interesting finding of the present study was the effect of the midline shift caused by tipping (UTS). Our results indicate a higher threshold for this type of midline shift (4 grad), which suggests that a shift caused by tipping has a less detrimental effect on the overall attractiveness when compared to a bodily shifted midline. The assessment of the need for treatment must be separated from the overall attractiveness. Regarding the treatment need, the assessments of the DP and LP almost corresponded; both groups considered a threshold of 3 mm for the midline shift in the male model. In the female model, however, the DP saw the need for treatment 1 mm earlier. Our results for the LP group agree with those of similar articles [10,19]. The lower threshold of the DP for the midline shifts could be justified by their training in this field. This discrepancy between the clinicians’ and patients’ perceptions plays a significant role in recognizing treatment need and treatment planning. Prioritizing patients’ perceptions of attractiveness and treatment need could help avoid over-treatment and reduce the costs and patient dissatisfaction.

## 5. Limitations

Our study’s demographic scope for both models and participants was quite narrow, adversely affecting the generalizability of our results. To eliminate potential bias from increased awareness of dental aesthetics, we excluded participants in the LP group with a history of aesthetic or orthodontic dental treatment in their close family. However, this evaluation relied on self-reports from the participants, which might not completely eliminate bias.

Despite our efforts, the LP group may have developed increased aesthetic dental awareness due to social media exposure. Given the rising popularity of social media among young adults in Germany, this exposure likely reflects the average dental awareness in society rather than individual history of dental aesthetic treatment [24]. It is reasonable to expect a higher aesthetic awareness in young adults in general.

Another limitation is the lack of replicability and validation of the methods used in this study. Future research is necessary to address these methodological concerns and to further explore this topic.

## 6. Conclusions

The results of this study showed that a dental midline shift significantly impacts perceived facial and smile attractiveness. The female model was judged more critically by all participants, regardless of their age or gender. Although the DP group had stricter standards for overall attractiveness, both the DP and LP groups showed similar assessments of treatment needs, with the DP group identifying the need for treatment at a lower threshold. These findings highlight the influence of professional background on aesthetic evaluations.

## Figures and Tables

**Table 1:** Effects of midline shifts on the perceived attractiveness of the models according to DP and LP groups.

Midline Shift	First Registration of a Decrease in the Smile’s Attractiveness			
	LP Group		DP Group	
	Female Model	Male Model	Female Model	Male Model
UBS (mm)	2	3	1	2
UTS (mm)	3	4	4	4
Total number of observers	40		40	

## Data Availability

The data presented in this study are available on request from the corresponding author.
